# Supplementary material for: A post-transcriptional program coordinated by CSDE1 prevents intrinsic neural differentiation of human embryonic stem cells
Source: Nat Commun. 2017 Nov 13;8:1456. doi: 10.1038/s41467-017-01744-5 (PMC5682285; doi:10.1038/s41467-017-01744-5)
Supplement: Supplementary file 1 — Supplementary Information [file 41467_2017_1744_MOESM1_ESM.pdf]

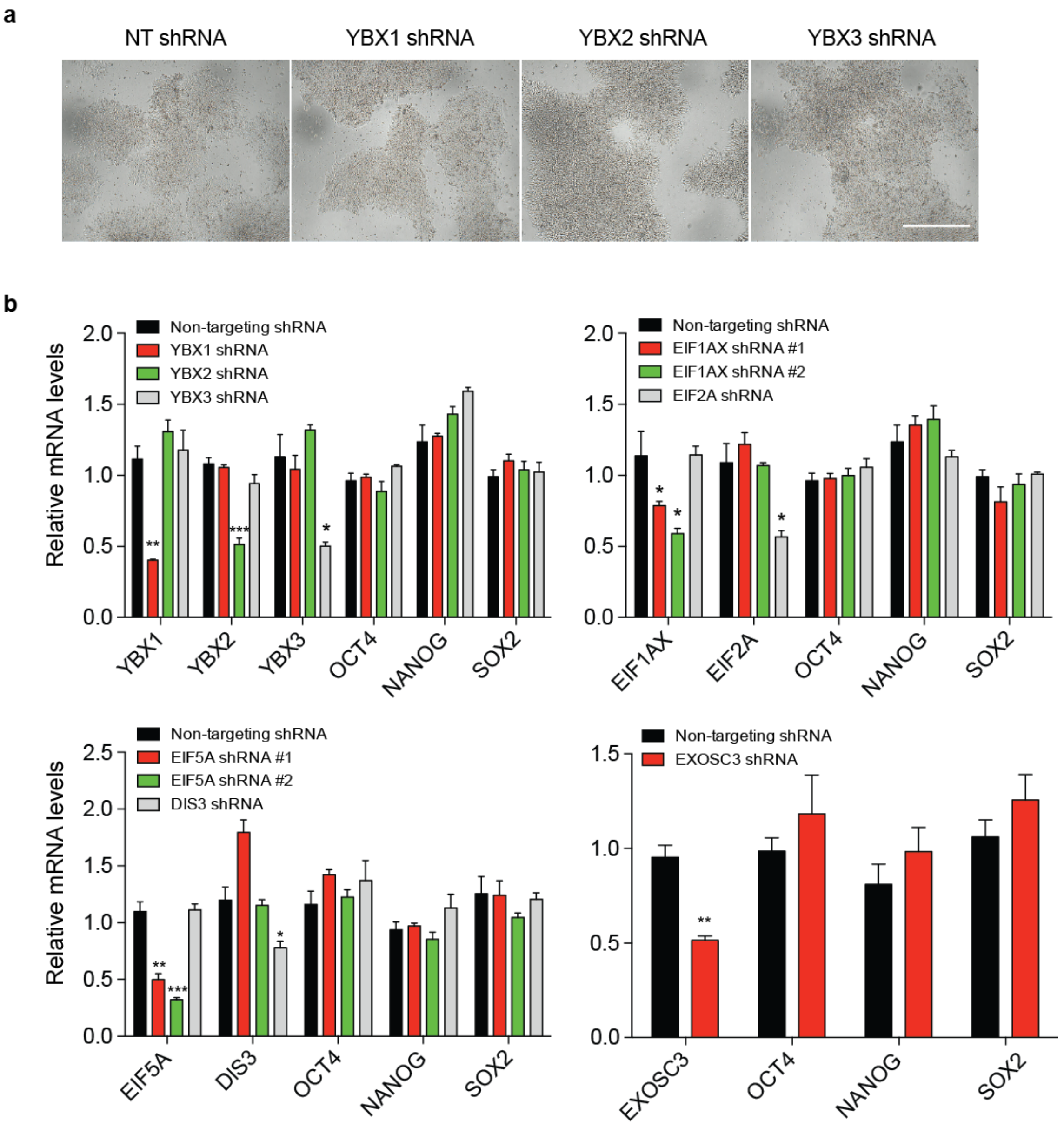

Supplementary Figure 1. shRNA screen against CSD-containing proteins in hESCs. (**a**) Brightfield images of H9 hESCs. Knockdown of YBX1, YBX2 and YBX3 do not change hESC colony morphology. Scale bar represents 500 µm. (**b**) Data represent the mean ± s.e.m. of mRNA relative expression levels to non-targeting (NT) shRNA H9 hESCs (n= 3 independent experiments). All the statistical comparisons were made by Student's t-test for unpaired samples. P-value: *(P<0.05), **(P<0.01), ***(P<0.001).

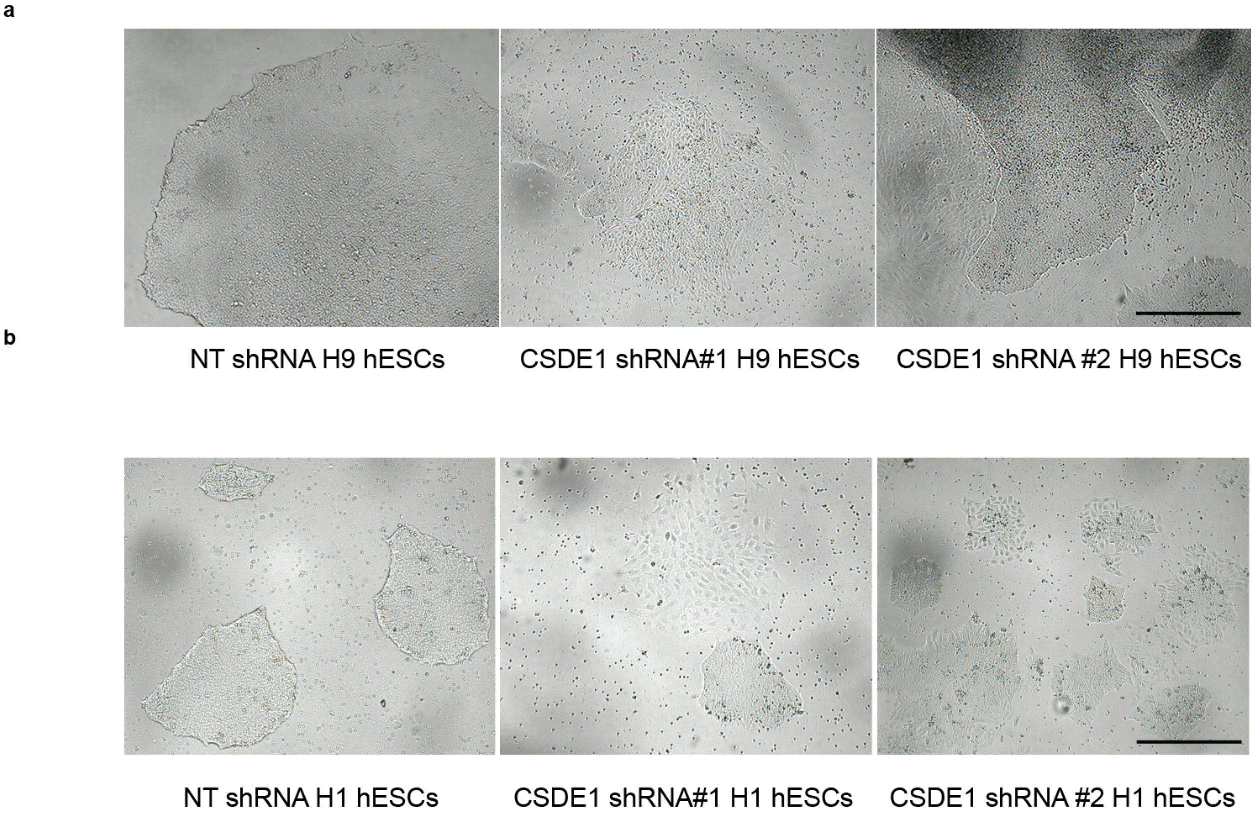

Supplementary Figure 2. Loss of CSDE1 promotes differentiation of hESCs. Brightfield images of H9 (**a**) and H1 (**b**) hESCs. Scale bars represent 500 μm.

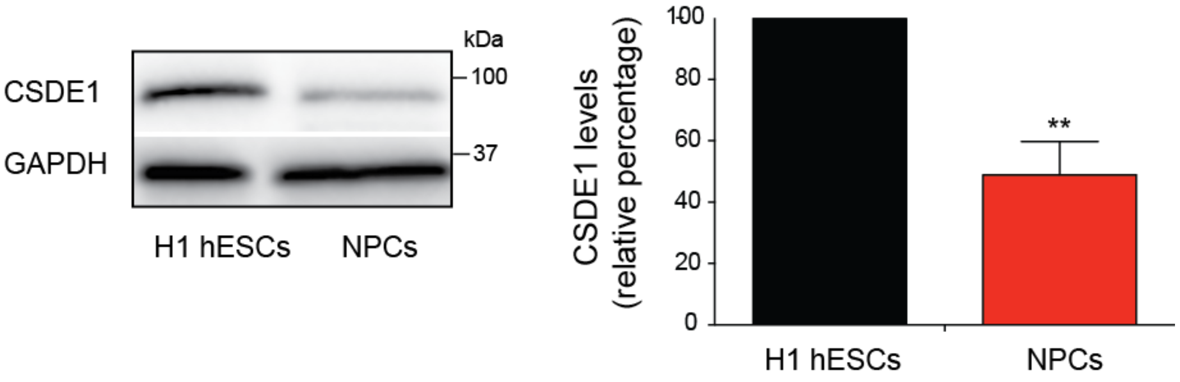

Supplementary Figure 3. CSDE1 protein levels decrease during neural differentiation of H1 hESCs. Western blot analysis with antibody to CSDE1. GAPDH is the loading control. The graph represents the CSDE1 relative percentage values (corrected for GAPDH loading control) to H1 hESCs (mean ± s.e.m. of three independent experiments).

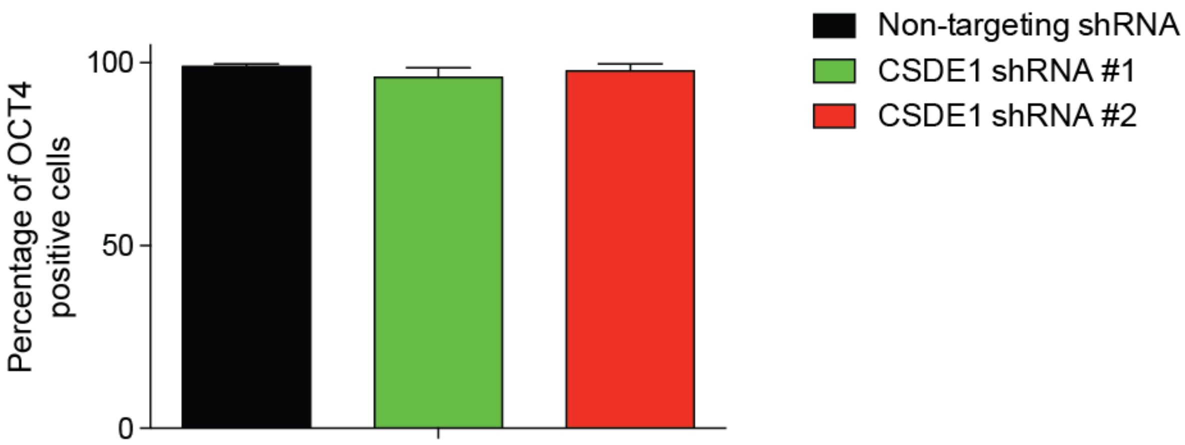

Supplementary Figure 4. CSDE1 KD hESC cultures are maintained by transferring typical undifferentiated colonies followed by daily monitoring to remove differentiated cells. Under this protocol, we did not observe differences in the percentage of OCT4 and PAX6-positive cells/total nuclei (mean ± s.e.m. of three independent experiments, 200 total cells per experiment). Statistical comparisons were made by Student's t-test for unpaired samples. On the contrary, when we grew hESCs without removing differentiated colonies, flattened cells proliferated in CSDE1 KD lines resulting in decreased percentage of OCT4-positive cells as compared with control hESC (please see **Fig. 2e-f**)

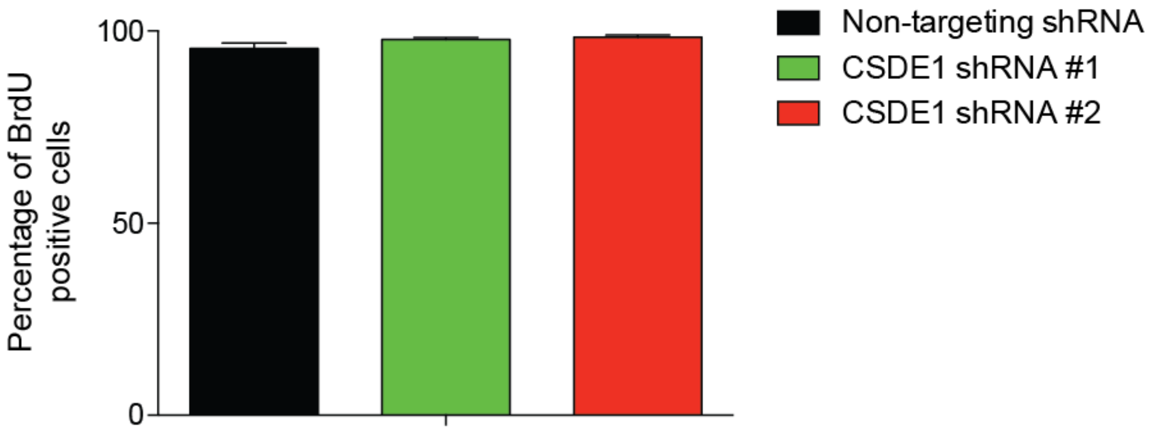

Supplementary Figure 5. Bromodeoxyuridine (BrdU) proliferation assay. Loss of CSDE1 does not induce significant differences in the percentage of BrdU positive cells (H9 NT hESCs *vs* CSDE1 shRNA#1 hESCs (P= 0.13), H9 NT hESCs *vs* CSDE1 shRNA #2 hESCs (P= 0.08)). The results are represented as averages of at least 1400 cells scored in six different experiments. Statistical comparisons were made by Student's t-test for unpaired samples.

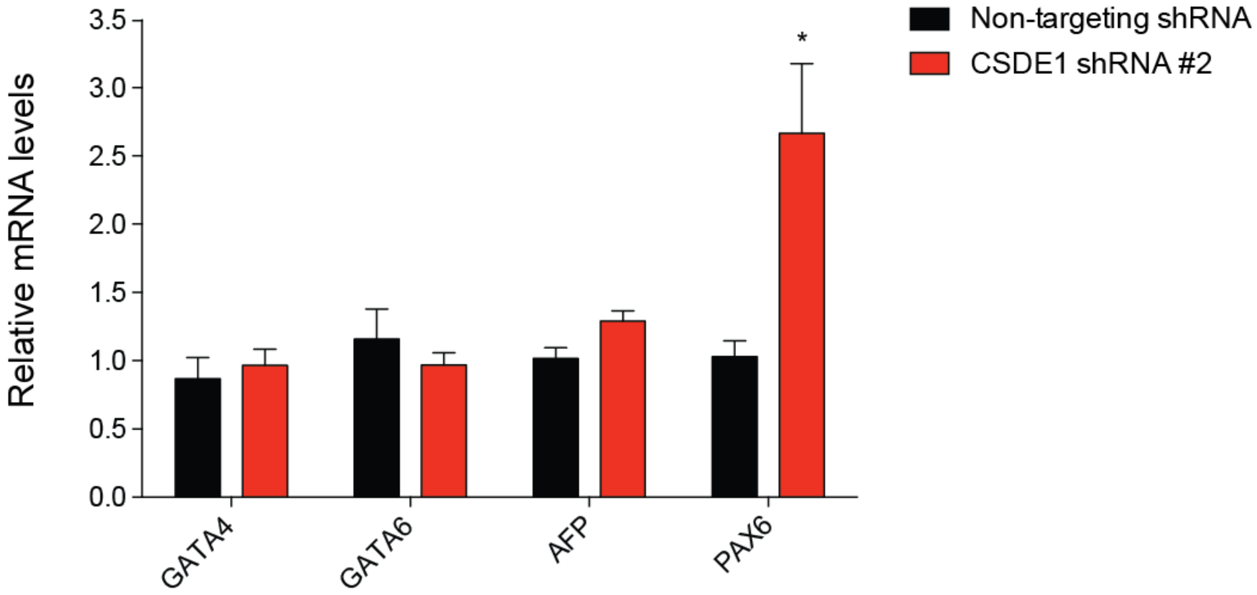

Supplementary Figure 6. Loss of CSDE1 does not trigger the expression of endoderm markers in hESCs. Quantitative PCR (qPCR) analysis of hESCs cultured for five days without removing differentiated cells. Graph (relative expression to non-targeting shRNA) represents the mean ± s.e.m. of three independent experiments. Statistical comparisons were made by Student's t-test for unpaired samples (P-value: *(P<0.05)).

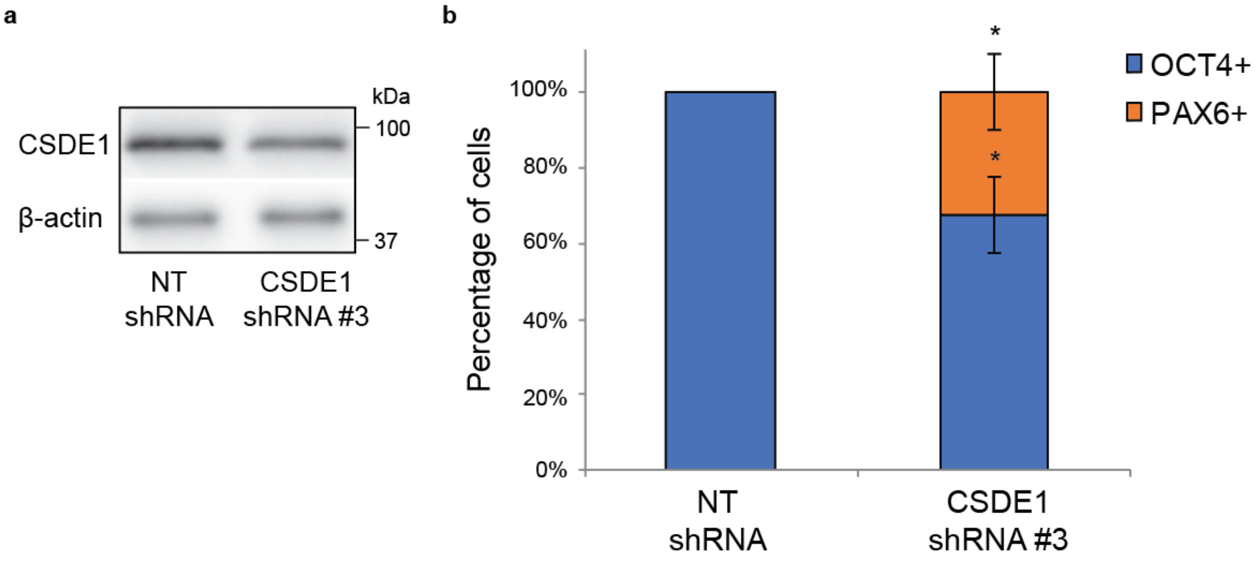

Supplementary Figure 7. Knockdown of CSDE1 with a shRNA to the 3'UTR region of the *CSDE1* transcript also induces spontaneous proliferation of PAX6-positive cells. (**a**) Western blot analysis with antibodies to CSDE1 in H9 hESC lysates. β-actin is the loading control. CSDE1 shRNA #3 targets the 3'UTR region of *CSDE1* transcript whereas the shRNAs #1 and #2 used in **Fig. 2e** target the coding region. (**b**) Percentage of OCT4 and PAX6-positive cells/total nuclei after five days without removing differentiated cells (mean ± s.e.m. of three independent experiments, 470-870 total cells per experiment). Statistical comparisons were made by Student's t-test for unpaired samples (P-value: *(P<0.05)).

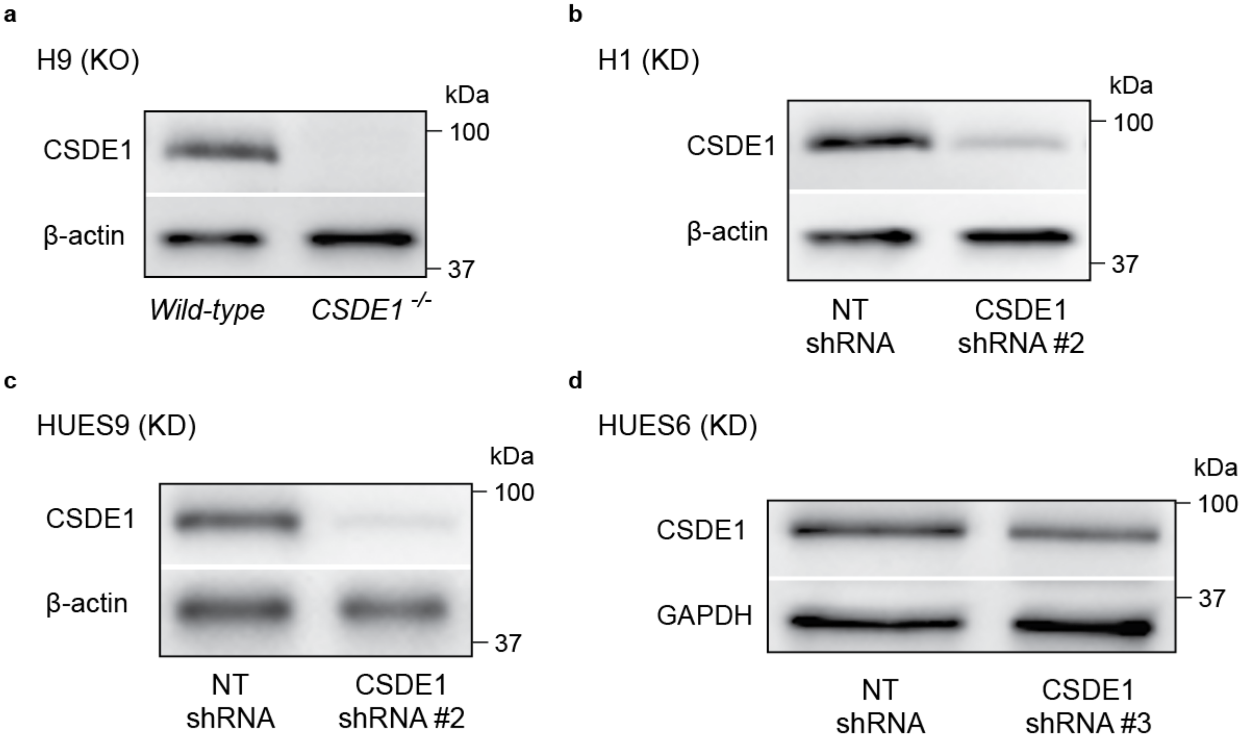

Supplementary Figure 8. CSDE1 protein expression levels in distinct knockout and knockdown hESC lines. Western blot analysis with antibodies to CSDE1 in (**a**) H9, (**b**) H1, (**c**) HUES9 and (**d**) HUES6 hESC lysates. As a loading control, we used β-actin (**a-c**) or GAPDH (**d**).

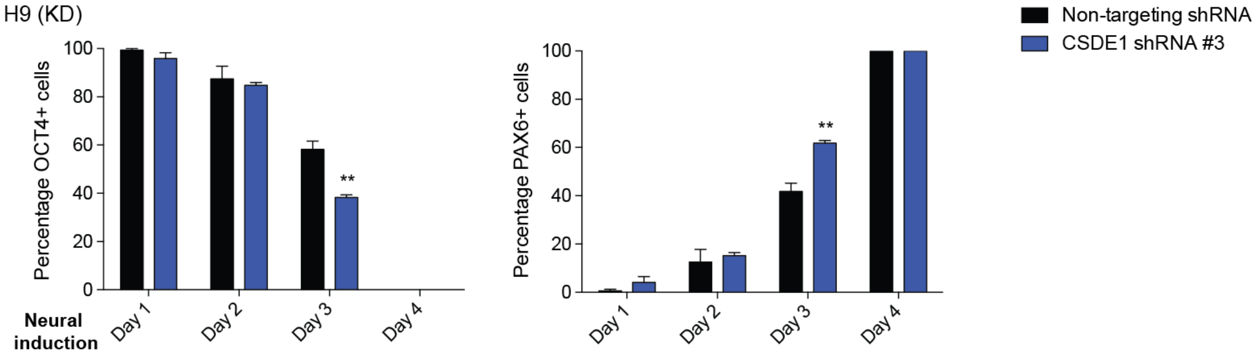

Supplementary Figure 9. Knockdown of CSDE1 with a shRNA to the 3'UTR region of the *CSDE1* transcript also accelerates neural differentiation. Percentage of OCT4 and PAX6-positive cells/total nuclei at different days after neural induction treatment of undifferentiated H9 hESCs (mean ± s.e.m. (n= 3, 400-500 total cells per data point). Statistical comparisons were made by Student's t-test for unpaired samples. P-value: **(P<0.01).

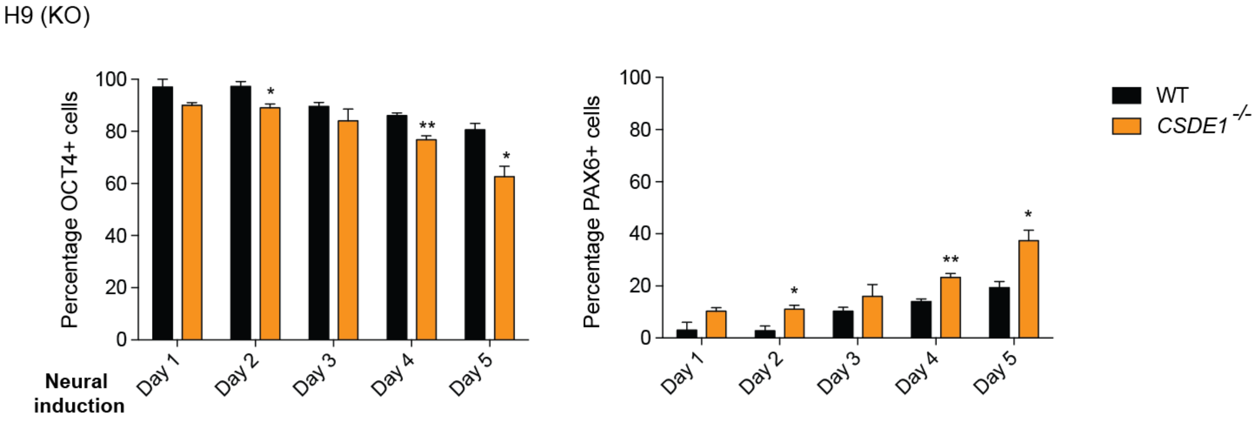

Supplementary Figure 10. Knockout of CSDE1 accelerates neural differentiation. Percentage of OCT4 and PAX6-positive cells/total nuclei at different days after neural induction treatment of undifferentiated H9 hESC colonies (mean ± s.e.m. of three independent experiments, 300-500 total cells per data point). Statistical comparisons were made by Student's t-test for unpaired samples. P-value: *(P<0.05), **(P<0.01). It is important to note that we observed a slower differentiation rate into PAX6-positive cells in these differentiation experiments compared to **Figure 3a**. Although neural induction was performed using H9 hESCs in both set of experiments, the control non-targeting shRNA as well as CSDE1 shRNA H9 lines used in **Figure 3a** were treated with puromycin every 3-4 days to select shRNA-expressing cells. On the contrary, both control wild-type (WT) and *CSDE1*$^{-/-}$ H9 hESCs were not maintained under regular puromycin treatments. This could contribute to the different rates observed in both neural induction experiments.

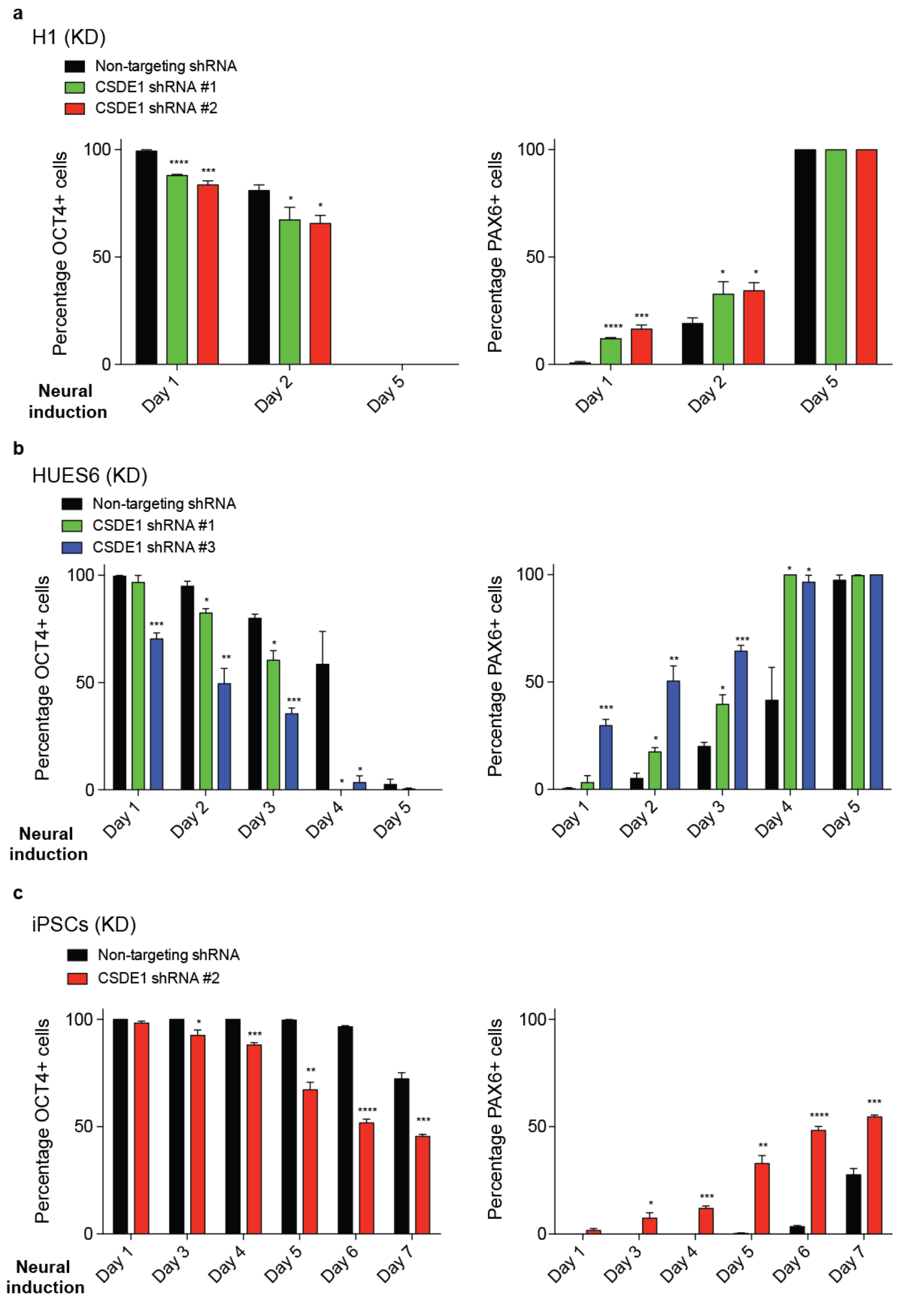

Supplementary Figure 11. Loss of CSDE1 promotes neural differentiation of distinct hESC lines and iPSCs. (**a**) Percentage of OCT4 and PAX6-positive cells/total nuclei at different days after neural induction of undifferentiated H1 hESCs (mean ± s.e.m. of three independent experiments, approximately 200 total cells per experiment). (**b**) Percentage of OCT4 and PAX6-positive cells/total nuclei at different days after neural induction of undifferentiated HUES6 hESCs (mean ± s.e.m., n= 3, 400-600 total cells per data point). (**c**) Percentage of OCT4 and PAX6-positive cells/total nuclei at different days after neural induction of undifferentiated iPSCs (mean ± s.e.m. of three independent experiments, 1000-1200 total cells per data point). Statistical comparisons were made by Student's t-test for unpaired samples. P-value: *(P<0.05), **(P<0.01), ***(P<0.001), **** (P<0.0001).

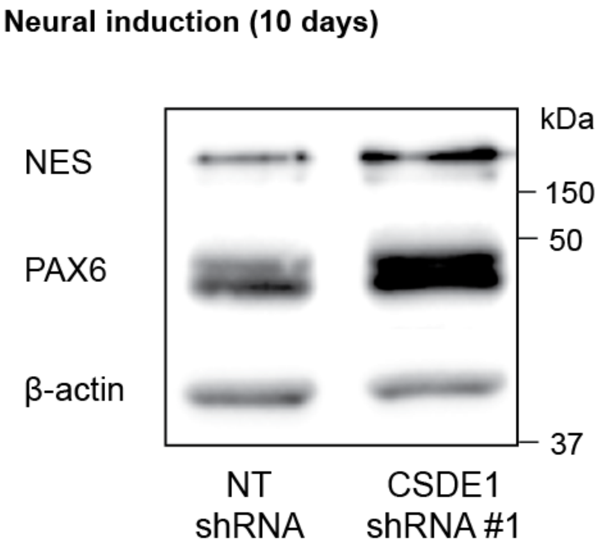

Supplementary Figure 12. CSDE1 KD cells exhibit increased protein levels of NPC markers. Western blot analysis of H9 cells after 10 days of neural induction treatment with antibodies to PAX6 and NES. β-actin is the loading control.

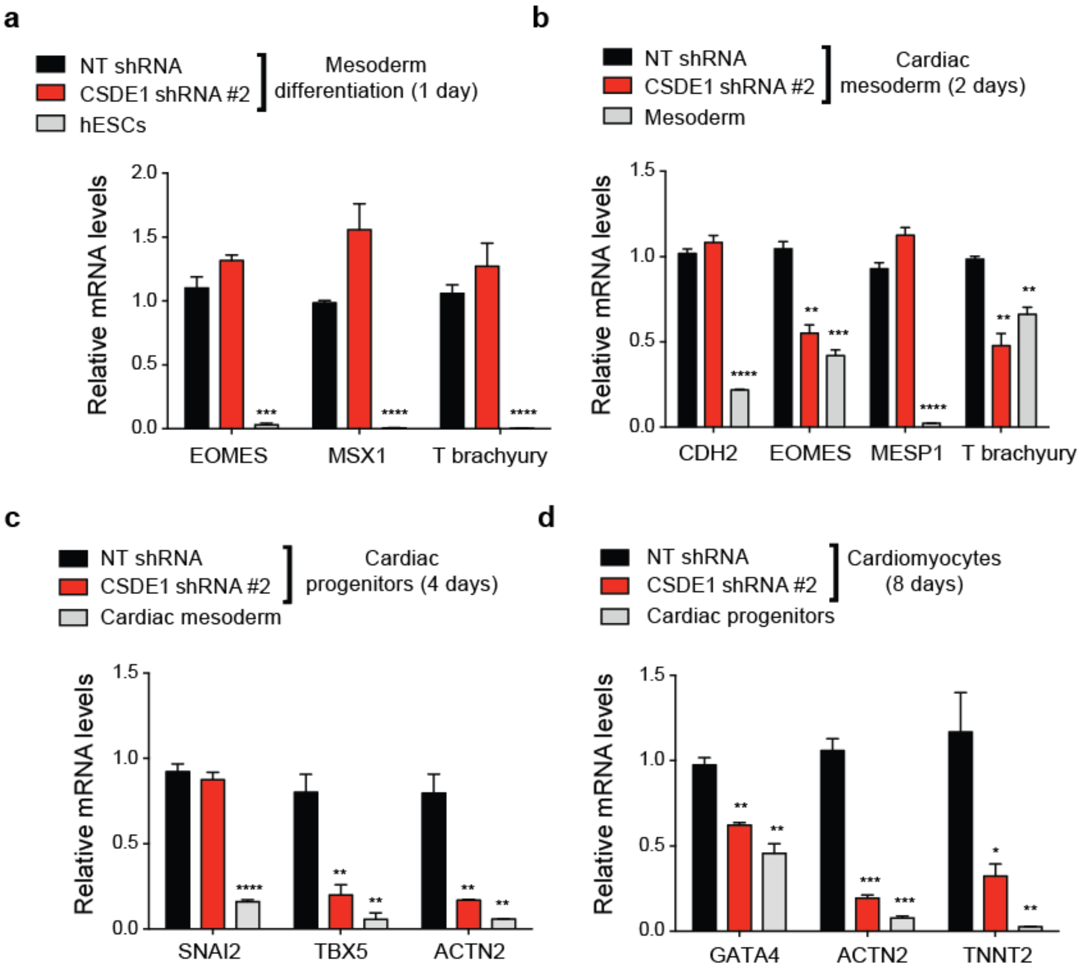

Supplementary Figure 13. CSDE1 KD hESCs exhibit diminished induction of cardiac markers during their differentiation into cardiomyocytes. (**a-d**) qPCR analysis at distinct stages of cardiomyocyte differentiation from H1 hESCs. Specific markers for each differentiation stage are shown in the corresponding graph. (**a**) Graph (relative expression to NT shRNA mesoderm) represent the mean ± s.e.m. (n= 3). (**b**) Graph (relative expression to NT shRNA cardiac mesoderm) represent the mean ± s.e.m. (n= 3). (**c**) Graph (relative expression to NT shRNA cardiac progenitors) represent the mean ± s.e.m. (n= 3). (**d**) Graph (relative expression to NT shRNA cardiomyocytes) represent the mean ± s.e.m. (n= 3). All the statistical comparisons were made by Student's t-test for unpaired samples. P-value: *(P<0.05), **(P<0.01), ***(P<0.001), **** (P<0.0001).

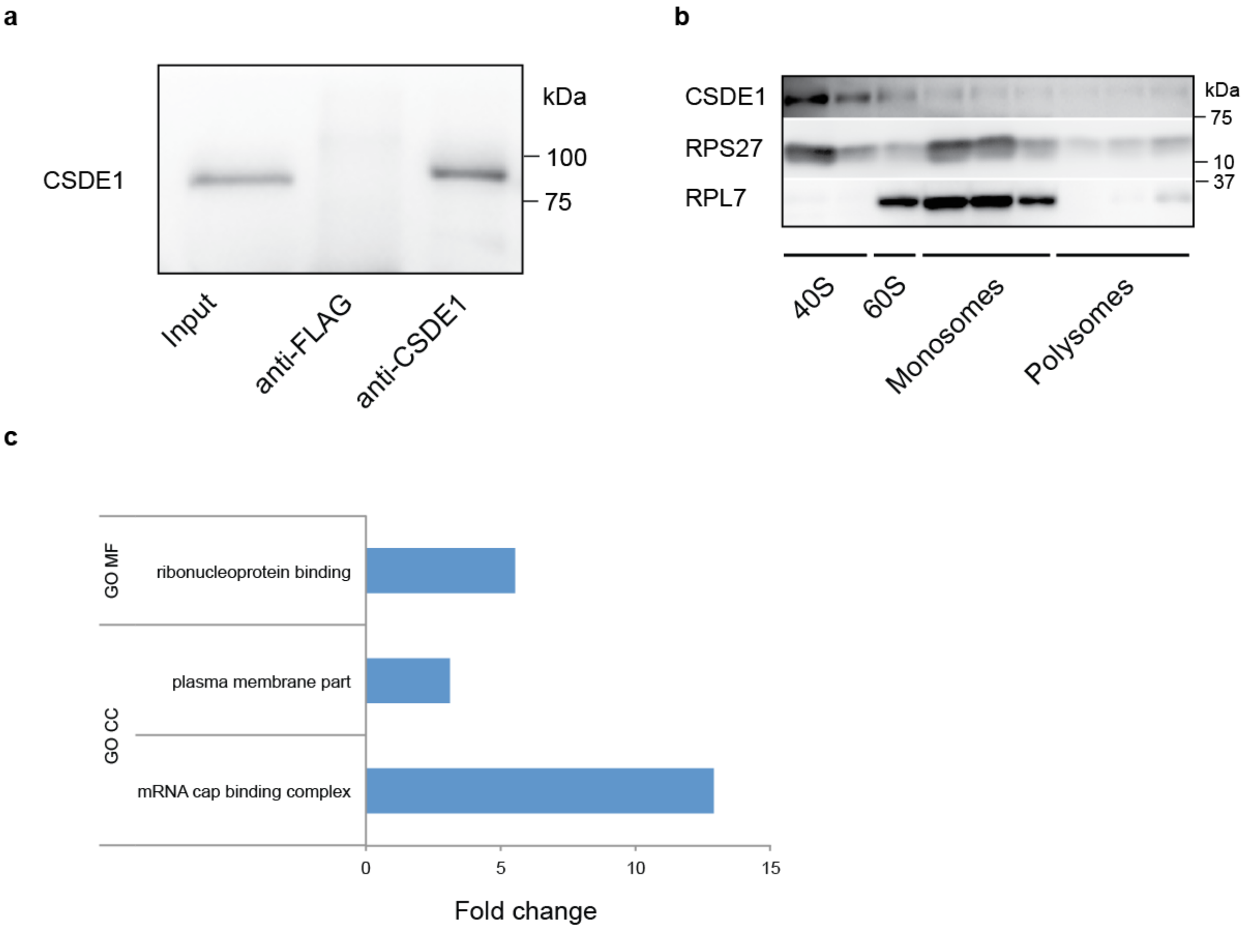

Supplementary Figure 14. CSDE1 binds to complexes involved in mRNA translation and stability in hESCs. (**a**) Co-immunoprecipitation with CSDE1 and FLAG antibodies followed by western blot with CSDE1 antibody in wild-type H9 hESC lysates. (**b**) Polysome profiling experiments followed by western blot with antibodies to CSDE1, RPS27 (40S ribosomal subunit) and RPL7 (60S ribosomal subunit). (**c**) Gene Ontology (GO) Cellular Component (CC) and GO Molecular Function (MF)-terms significantly enriched after co-IP experiments with CSDE1 antibody (as defined by cut-off t-test Difference >2, p-value <0.02).

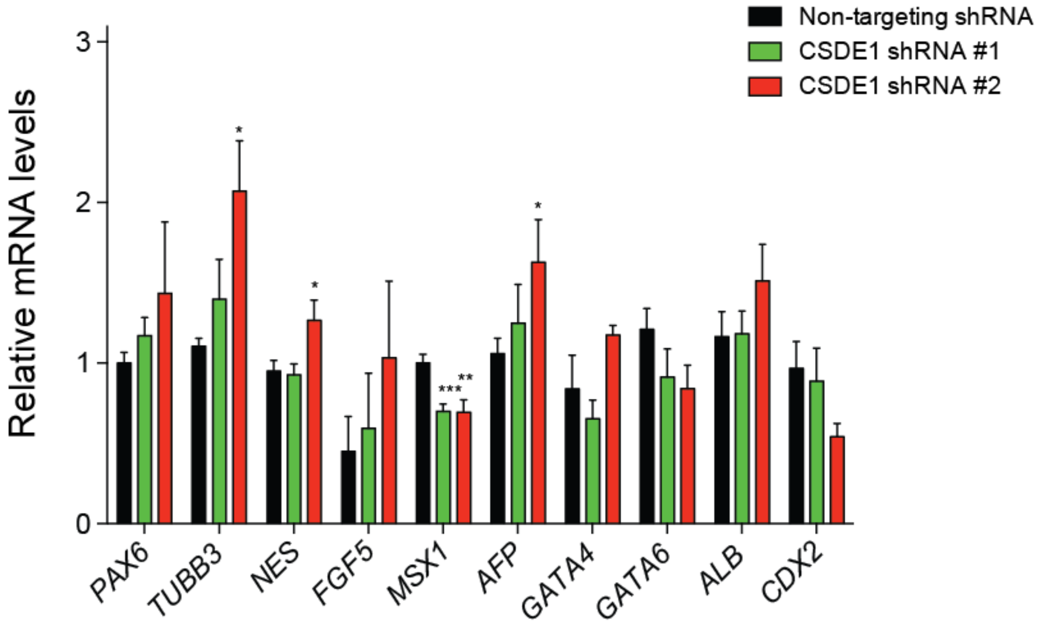

Supplementary Figure 15. Analysis of germ layer markers in CSDE1 KD H9 hESC cultures primarily formed by undifferentiated colonies. qPCR analysis of ectodermal (*PAX6*, *NES*, *FGF5*), mesodermal (*MSX1*) and endodermal (*ALB*, *GATA4*, *GATA6*) germ layer markers in hESCs cultures daily monitored to remove differentiated cells. Graph (relative expression to NT shRNA) represents the mean ± s.e.m. of four independent experiments with three biological replicates. Statistical comparisons were made by Student's t-test for unpaired samples. P-value: *(P<0.05), **(P<0.01), ***(P<0.001).

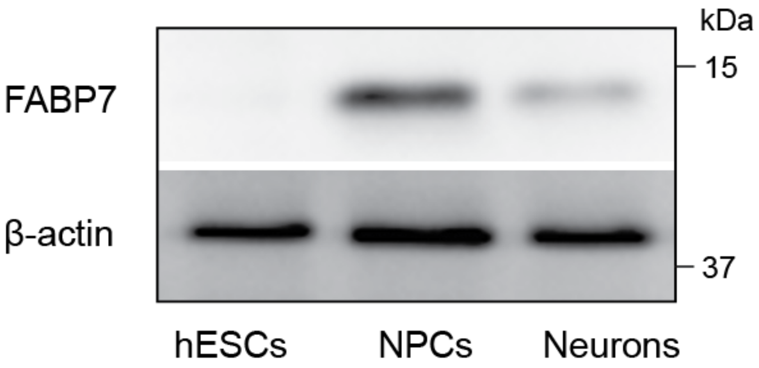

Supplementary Figure 16. FABP7 is highly expressed in NPCs derived from hESCs. Western blot analysis of H9 hESCs and their differentiated counterparts with antibody to FABP7. β-actin is the loading control.

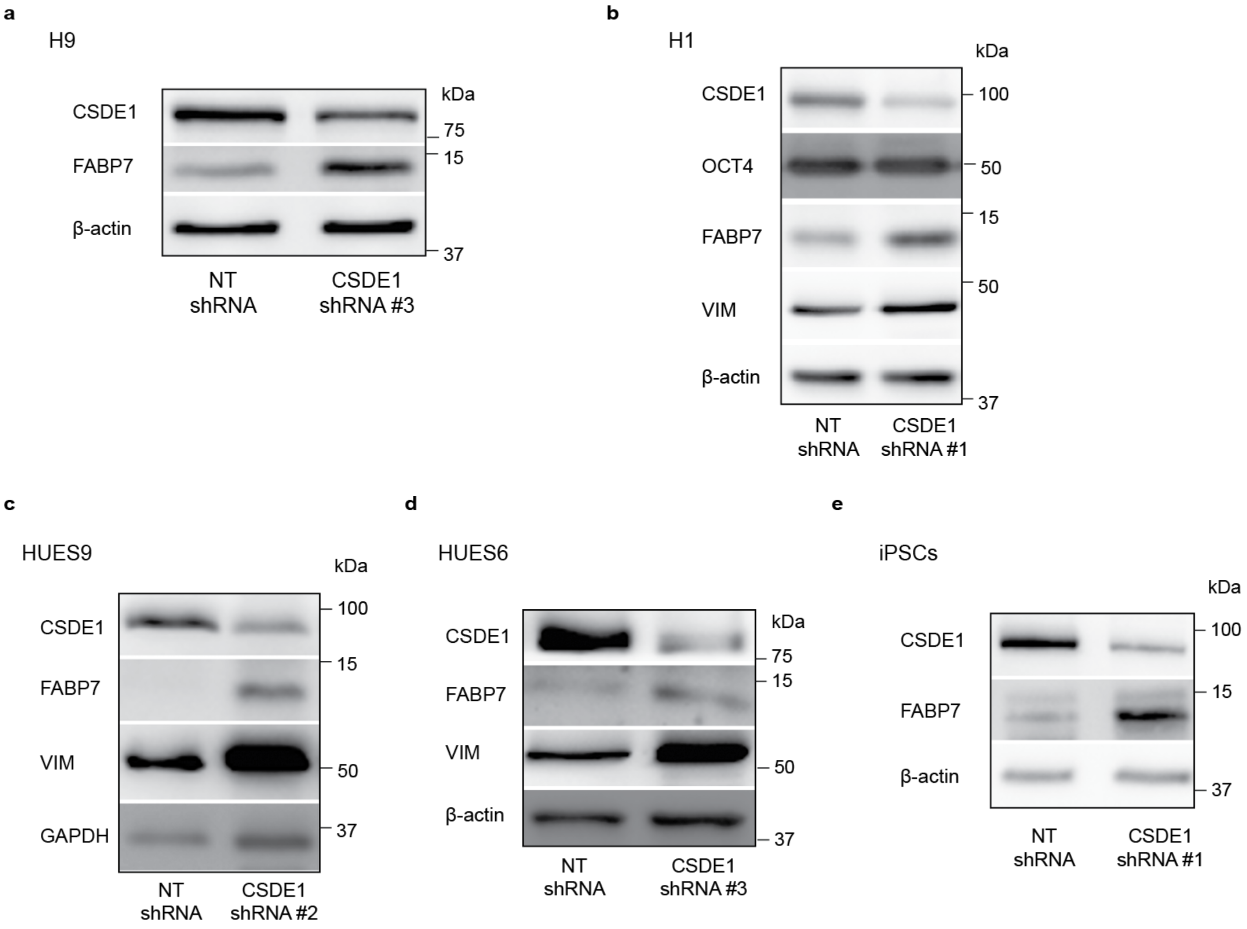

Supplementary Figure 17. Loss of CSDE1 results in increased levels of FABP7. Western blot analysis in (**a**) H9 hESC, (**b**) H1 hESC, (**c**) HUES9 hESC, (**d**) HUES6 hESC and (**e**) iPSC lysates. As a loading control, we used β-actin (**a, b, d, e**) or GAPDH (**c**).

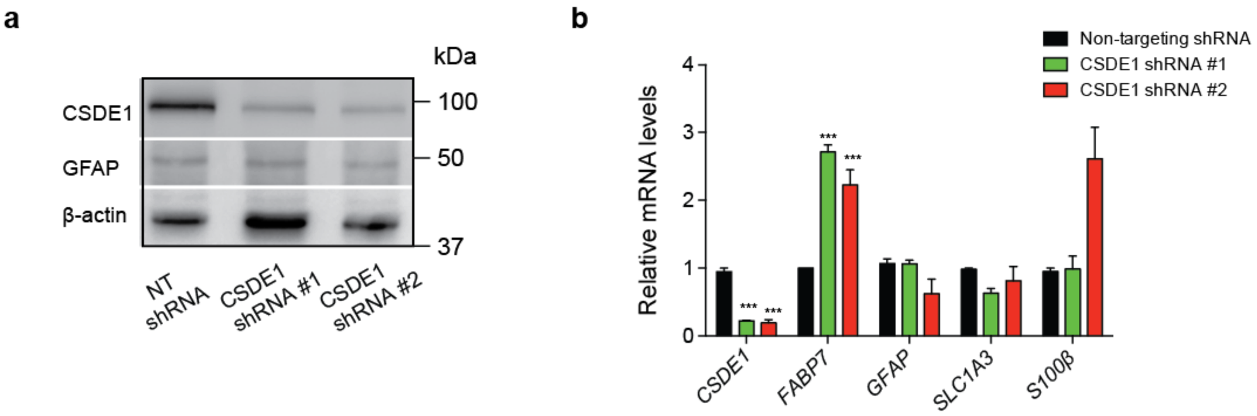

Supplementary Figure 18. Loss of CSDE1 does not alter the levels of radial glia markers such as GFAP, SLC1A3 or S100ß. (**a**) Western blot analysis of H9 hESCs with antibodies to GFAP. β-actin is the loading control. (**b**) Graph (relative expression to NT shRNA H9 hESCs) represents the mean ± s.e.m. of two independent experiments with three biological replicates. All the statistical comparisons were made by Student's t-test for unpaired samples. P-value: ***(P<0.001).

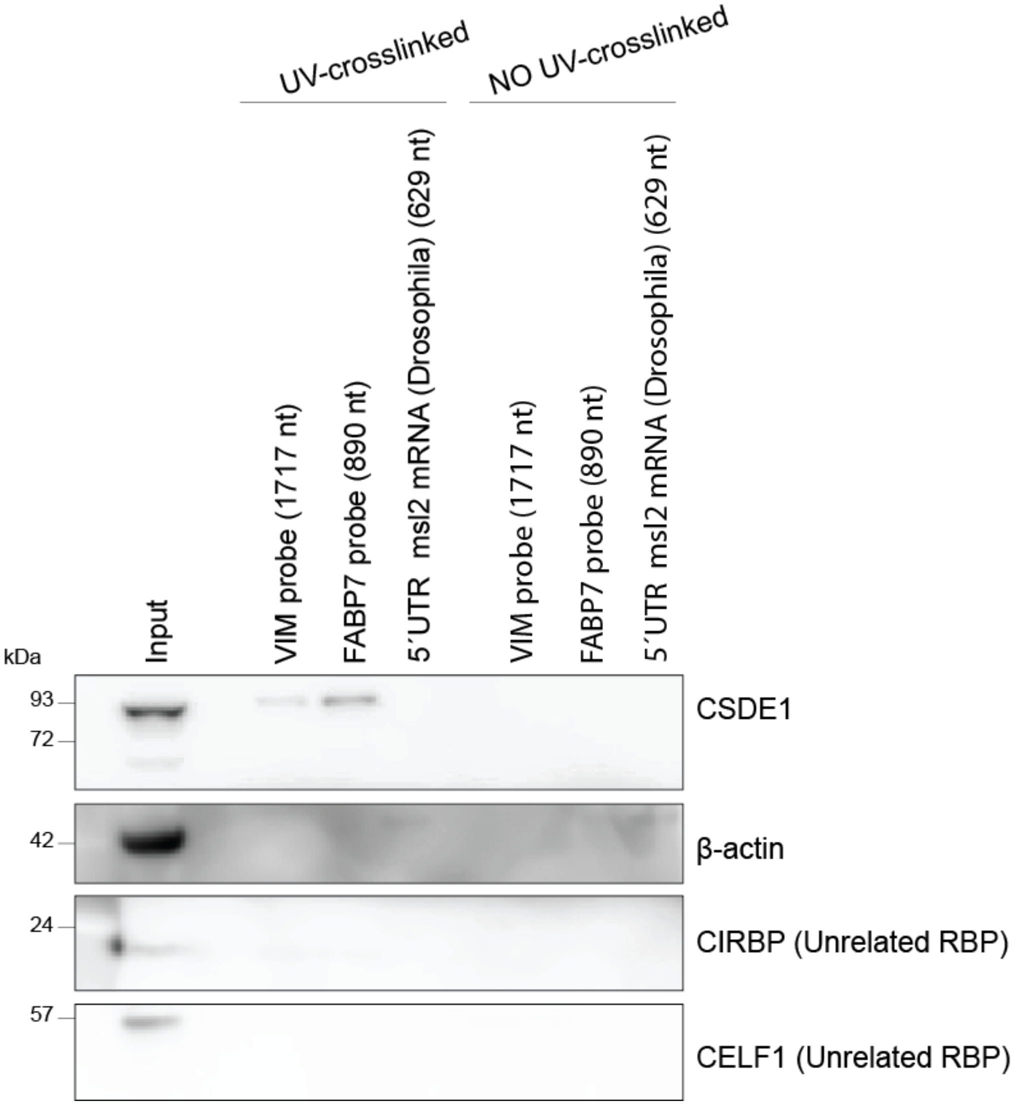

Supplementary Figure 19. VIM and FABP7 RNA probes pull down CSDE1 protein from H9 hESC lysates. Pull-down assay using *in vitro* transcribed biotinylated *VIM*, *FABP7* and 5´UTR *msl2* RNA probes (negative control). RNA pull-down specificity was assessed by Western blotting using antibodies to CSDE1, unrelated RBPs (CIRBP and CELF1) and β-actin.

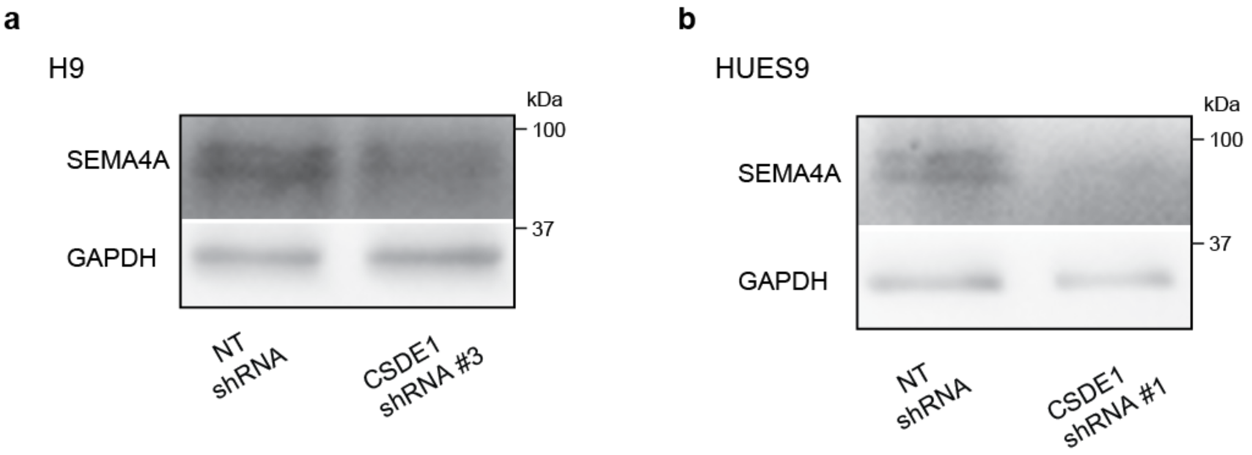

Supplementary Figure 20. Knockdown of CSDE1 results in decreased protein levels of SEMA4A. Western blot analysis of (**a**) H9 hESCs and (**b**) HUES9 hESCs with antibody to SEMA4A. GAPDH is the loading control.

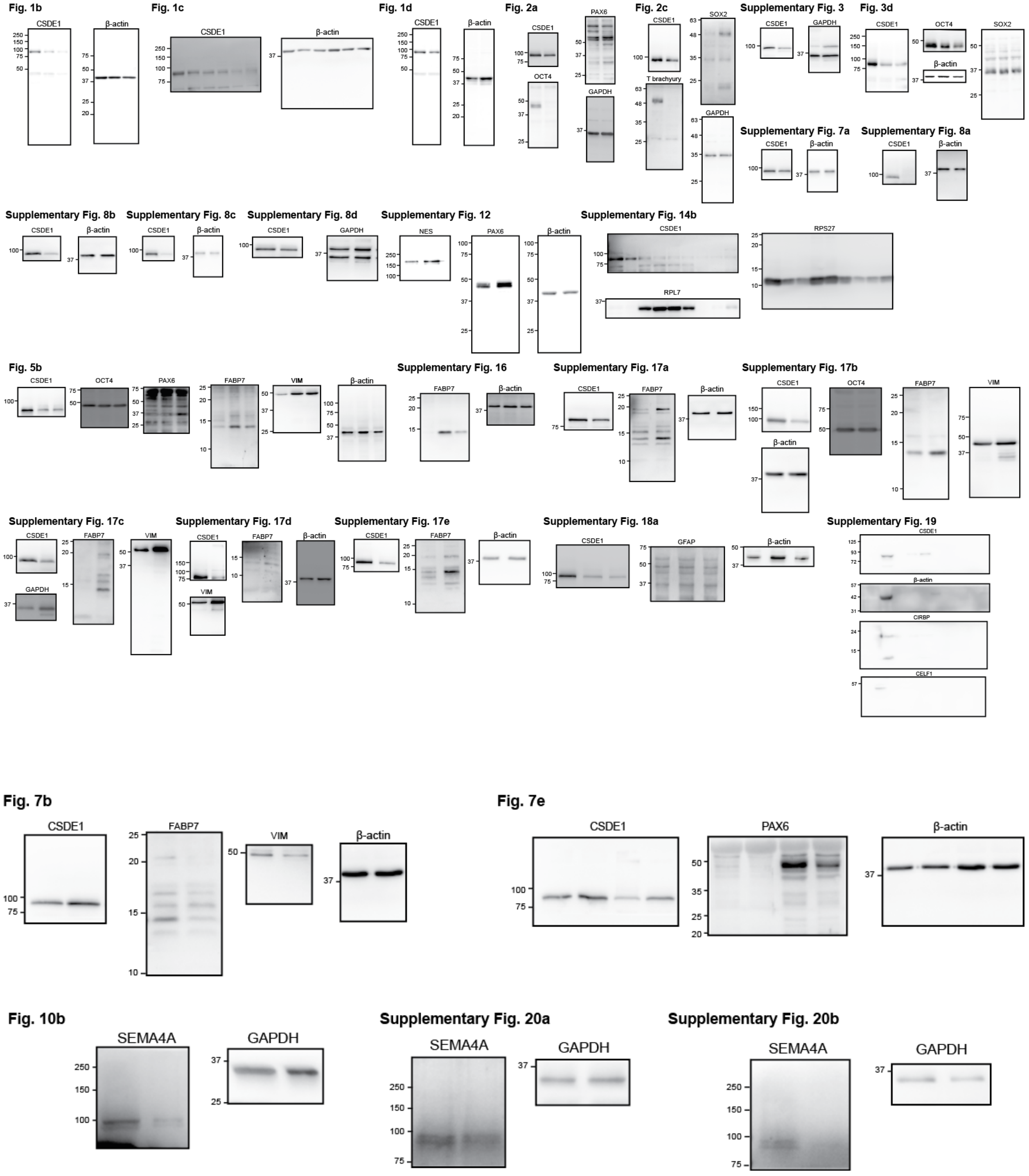

Supplementary Figure 21. Uncropped images of most important western blots. Uncropped images are presented with molecular weight ladders.

Gene names	Domain	hESC vs neurons ratio	q-value
LIN28A	**CSD**	15.26	0.033
LIN28B	**CSD**	Not detected	
YBX1	**CSD**	2.38	0.009
YBX2	**CSD**	2.40	0.013
YBX3	**CSD**	Not detected	
CSDE1	**CSD**	2.66	0.034
DIS3	**CSD and S1**	1.78	0.013
EIF1AX	**S1**	2.19	0.007
EIF2A	**S1**	Not detected	
EIF5A	**S1**	1.67	0.009
EXOSC3	**S1**	Not detected	
DHX8	**S1**	Not detected	

Supplementary Table 1. CSD and CSD-like proteins are increased in hESCs compared with neurons. Tandem mass tag (TMT) quantitative proteomics comparing H9 hESCs with their differentiated neuronal counterparts. CSD and CSD-like proteins are significantly increased in hESCs. Statistical comparisons were made by Student's t-test (n= 3). False Discovery Rate (FDR) adjusted p-value (q-value) <0.05 was considered significant.

(a)

Gene names	Protein names
RPS27;RPS27L	40S ribosomal protein S27
SMNDC1	Survival of motor neuron-related-splicing factor 30
FAM103A1	RNMT-activating mini protein
SMC1A	Structural maintenance of chromosomes protein 1A
PES1	Pescadillo homolog
PUM1	Pumilio homolog 1
CHCHD6	MICOS complex subunit MIC25
HIST1H2BH;HIST2H2BF	Histone H2B type 1-H;Histone H2B type 2-F;Histone H2B
SSSCA1	Sjoegren syndrome/scleroderma autoantigen 1
PSMC2	26S protease regulatory subunit 7
GDI2;GDI1	Rab GDP dissociation inhibitor beta;Rab GDP dissociation inhibitor alpha
NCAPG	Condensin complex subunit 3
TAGLN2	Transgelin-2
NME1	Nucleoside diphosphate kinase A
PTMS	Parathymosin
G6PD	Glucose-6-phosphate 1-dehydrogenase
AMPD2	AMP deaminase 2
SAMM50	Sorting and assembly machinery component 50 homolog
MAP7	Ensconsin
REPS1	Calcium binding
ZNF512	TransriptionZinc finger protein 512

(b)

Gene names	Protein names	T-test difference	-log P-value
CSDE1	Cold shock domain-containing protein E1	10.47	3.50
STRAP	Serine-threonine kinase receptor-associated protein	9.63	2.96
SUGP2	SURP and G-patch domain-containing protein 2	6.86	3.81
IMMT	MICOS complex subunit MIC60	5.74	4.81

Supplementary Table 2. Protein interactors of CSDE1 in H9 hESCs. Protein label-free quantification (LFQ) values from co-immunoprecipitation (co-IP) experiments using CSDE1 antibody compared to control co-IP with FLAG antibody. (**a**) List of proteins only co-immunoprecipitated with CSDE1 antibody but not with FLAG antibody (n= 3). (**b**) List of proteins significantly increased after co-IP with CSDE1 compared with FLAG antibody (Student's t-test, n= 3, FDR< 0.05).

Gene names	Protein names	T-test difference		-log P-value	
		CSDE1 shRNA 1	CSDE1 shRNA 2	CSDE1 shRNA 1	CSDE1 shRNA 2
FABP7	Fatty acid-binding protein, brain	1.30	0.86	3.06	3.90
VIL1	Villin-1	1.10	0.84	2.14	1.71
FGFBP3	Fibroblast growth factor-binding protein 3	0.81	0.52	4.02	2.45
PAIP2	Polyadenylate-binding protein-interacting protein 2	0.80	0.40	2.01	2.31
FLNC	Filamin-C	0.78	0.72	2.54	2.11
S100A4	Protein S100-A4	0.70	1.07	2.18	2.71
VIM	Vimentin	0.66	0.33	4.88	2.71
CIZ1	Cip1-interacting zinc finger protein	0.58	0.60	2.21	1.62
DNM1	Dynamin-1	0.52	059	2.54	2.78
SMARCC2	SWI/SNF complex subunit SMARCC2	0.37	0.34	2.75	2.34
CSDE1	Cold shock domain-containing protein E1	-2.07	-2.52	4.62	5.76
VSNL1	Visinin-like protein 1	-1.18	-1.14	2.28	3.43
THUMPD3	THUMP domain-containing protein 3	-1.12	-2.09	1.83	2.59
EIF4A2	Eukaryotic initiation factor 4A-II	-1.09	-0.52	2.41	1.68
ENPP1	Alkaline phosphodiesterase I	-1.09	-0.54	2.43	1.59
PRPS1	Ribose-phosphate pyrophosphokinase 1	-0.73	-0.47	2.63	1.99
DCLK1	Serine/threonine-protein kinase DCLK1	-0.66	-0.48	2.54	2.68
SPG20	Spartin	-0.59	-0.99	1.92	2.39
ANXA2	Annexin A2	-0.54	-0.38	2.11	3.96
TBC1D15	TBC1 domain family member 15	-0.42	-0.57	3.14	3.63
PTBP2	Polypyrimidine tract-binding protein 2	-0.32	-0.45	3.56	2.42

Supplementary Table 3. List of significantly changed proteins in quantitative proteomic analysis of CSDE1 KD H9 hESCs. Knockdown of CSDE1 induces an increase in the levels of FABP7 and VIM. Means are calculated from the log2 of LFQ values (LFQ CSDE1 shRNA hESCs/Non-targeting shRNA hESCs). Statistical comparisons were made by Student's t-test (n= 3, FDR< 0.15).
