# Supplementary material for: A post-transcriptional program coordinated by CSDE1 prevents intrinsic neural differentiation of human embryonic stem cells
Source: Nat Commun. 2017 Nov 13;8:1456. doi: 10.1038/s41467-017-01744-5 (PMC5682285; doi:10.1038/s41467-017-01744-5)
Supplement: Supplementary file 3 — Description of Additional Supplementary Files [file 41467_2017_1744_MOESM3_ESM.pdf]

Description of Supplementary Files

File name: Supplementary Data 1
Description: Tandem mass tag (TMT) quantitative proteomics data comparing hESCs with their differentiated neuronal counterparts. Statistical comparisons were made by Student's t-test (n= 3). False Discovery Rate (FDR) <0.05 was considered significant.

File name: Supplementary Data 2
Description: Protein label-free quantification (LFQ) values from co-immunoprecipitation (co-IP) experiments using CSDE1 antibody compared to control co-IP with FLAG antibody. Statistical comparisons were made by Student's t-test (n= 3). FDR< 0.05 was considered significant.

File name: Supplementary Data 3
Description: Quantitative proteomic analysis of CSDE1 KD H9 hESCs. Means are calculated from the log2 of LFQ values (LFQ CSDE1 shRNA hESCs/Non-targeting shRNA hESCs). Statistical comparisons were made by Student's t-test (n= 3, FDR< 0.15).

File name: Supplementary Data 4
Description: Number of CSDE1 consensus binding motifs identified in transcripts showing altered levels upon CSDE1 knockdown from RNA sequencing experiments.

File name: Supplementary Data 5
Description: Analysis of transcriptomic data from CSDE1 KD H9 hESCs compared to non-targeting (NT) shRNA control H9 hESCs. Transcripts showing a log2-fold change at a FDR <0.05 were retained as significantly differentially expressed.

File name: Supplementary Data 6
Description: Gene ontology (GO) biological process analysis of transcripts changed in CSDE1 KD hESCs.

File name: Supplementary Data 7
Description: Quantitative PCR analysis in independent hESC lines and iPSCs of potential CSDE1-regulated transcripts.

File name: Supplementary Data 8
Description: List of primers used for qPCR experiments.
